# Both RyRs and TPCs are required for NAADP-induced intracellular Ca^2+^ release

**DOI:** 10.1016/j.ceca.2015.05.005

**Published:** 2015-09

**Authors:** Julia V. Gerasimenko, Richard M. Charlesworth, Mark W. Sherwood, Pawel E. Ferdek, Katsuhiko Mikoshiba, John Parrington, Ole H. Petersen, Oleg V. Gerasimenko

**Affiliations:** a Medical Research Council Group, Cardiff School of Biosciences, Cardiff University, Cardiff, Wales, UK; b Laboratory for Developmental Neurobiology, Riken Brain Science Institute, Wako City, Saitama, Japan; c Ca^2+^ Oscillation Project, ICORP-SORST, JST, Wako City, Saitama, Japan; d Department of Pharmacology, University of Oxford, Mansfield Road, Oxford OX1 3QT, UK

**Keywords:** RyR, ryanodine receptor, TPC, two-pore channel, NAADP, nicotinic acid adenine dinucleotide phosphate, cADPR, cyclic-ADP-ribose, IP_3_, inositol trisphosphate, KO, knockout, ZG, zymogen granules, ER, endoplasmic reticulum, CCK, cholecystokinin, ACh, acetylcholine, Pancreatic acinar cells, TPC2 knockouts, cADPR, Acidic store, RyR3 knockouts

## Abstract

Intracellular Ca^2+^ release is mostly mediated by inositol trisphosphate, but intracellular cyclic-ADP-ribose (cADPR) and nicotinic acid adenine dinucleotide phosphate (NAADP) are important messengers in many systems. Whereas cADPR generally activates type 2 ryanodine receptors (RyR2s), the NAADP-activated Ca^2+^ release mechanism is less clear. Using knockouts and antibodies against RyRs and Two-Pore Channels (TPCs), we have compared their relative importance for NAADP-induced Ca^2+^ release from two-photon permeabilized pancreatic acinar cells. In these cells, cholecystokinin-elicited Ca^2+^ release is mediated by NAADP. TPC2-KO reduced NAADP-induced Ca^2+^ release by 64%, but the combination of TPC2-KO and an antibody against TPC1, significantly reduced Ca^2+^ release by 86% (64% vs. 86%, *p* < 0.0002). In RyR3-KO, NAADP-evoked Ca^2+^ release reduced by ∼50% but, when combined with antibodies against RyR1, responses were 90% inhibited. Antibodies against RyR2 had practically no effect on NAADP-evoked Ca^2+^ release, but reduced release in response to cADPR by 55%. Antibodies to RyR1 inhibited NAADP-induced Ca^2+^ liberation by 81%, but only reduced cADPR responses by 30%. We conclude that full NAADP-mediated Ca^2+^ release requires both TPCs and RyRs. The sequence of relative importance for NAADP-elicited Ca^2+^ release from the all stores is RyR1 > TPC2 > RyR3 > TPC1 >> RyR2. However, when assessing NAADP-induced Ca^2+^ release solely from the acidic stores (granules/endosomes/lysosomes), antibodies against TPC2 and TPC1 virtually abolished the Ca^2+^ liberation as did antibodies against RyR1 and RyR3. Our results indicate that the primary, but very small, NAADP-elicited Ca^2+^ release via TPCs from endosomes/lysosomes triggers the detectable Ca^2+^-induced Ca^2+^ release via RyR1 and RyR3 occurring from the granules and the ER.

## Highlights

• Antibody against RyR1 reduced NAADP-evoked Ca^2+^ release by 81%.
• Combined inhibition of RyR1 and RyR3 (or RyR3-KO) reduced responses to NAADP by >90%.
• Knockout of TPC2 (or antibody against TPC2) reduced responses to NAADP by 64%.
• Combined inhibition of TPC2 and TPC1 reduced responses by 86%.
• In acidic stores inhibition of either pair of RyR1/3 or TPC1/2 abolished responses.

## Graphical abstract

**Figure:**
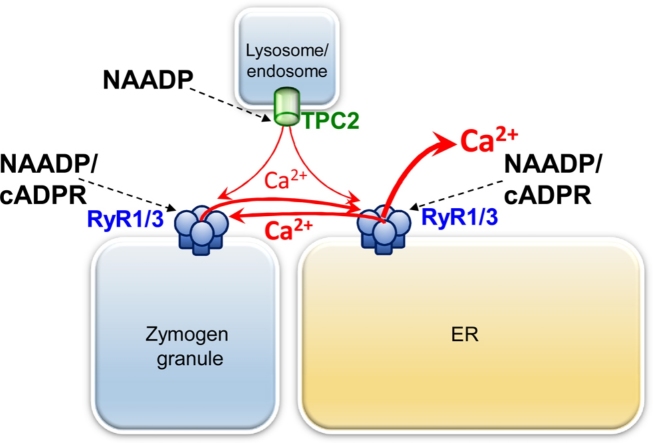

## 1 Introduction

Exocrine gland cells have provided the most important models for elucidating the mechanisms underlying hormone- or neurotransmitter-evoked intracellular Ca^2+^ release. Early work on salivary glands provided evidence for Ca^2+^ pump-mediated Ca^2+^ uptake into intracellular stores [1] as well as neurotransmitter-elicited liberation of Ca^2+^ from such stores [2]. Ca^2+^ signalling studies on pancreatic acinar cells led to many important findings including the discovery of inositol trisphosphate (IP_3_) as a messenger releasing Ca^2+^ from intracellular stores [3], localized Ca^2+^ signal generation in the apical granular pole of the cells [4–6] and intracellular Ca^2+^ tunnels [7]. It has been shown that IP_3_ induces responses from the endoplasmic reticulum ER [8], suggesting that IP_3_ receptors (IP_3_Rs) are located in the ER including the apical area of acinar cells [8–10]. However, it was also found that IP_3_ can release Ca^2+^ from a different organelle containing a vacuolar H^+^-ATPase [11] followed up by the discovery of IP_3_-evoked Ca^2+^ release from bovine adrenal medullary secretory vesicles [12]. Work on isolated pancreatic zymogen granules (ZGs) demonstrated directly both IP_3_ and cADPR-induced Ca^2+^ release from this organelle [13] and was later confirmed by a study of tracheal goblet cells [14]. Apart from the ZGs, other acid organelles (endosomes and lysosomes) can also release Ca^2+^ [15–17]. Under normal physiological conditions the apical Ca^2+^ spikes regulating exocytotic secretion of digestive enzymes are mainly due to Ca^2+^ release from the ER strands in the secretory granular area [18], which are functionally connected to the lumen of the bulk of the ER, allowing diffusion of Ca^2+^ inside the ER throughout the cell [7,19]. In contrast, release of Ca^2+^ from the acid stores may be of particular significance for the initiation of acute pancreatitis [20,21]. The acid stores, like the ER, have been shown to respond to all the three intracellular Ca^2+^ releasing messengers IP_3_, cADPR and NAADP [18,22]. Previous work has emphasized the importance of the RyRs for the action of CCK, which is primarily mediated by NAADP, in contrast to the action of acetylcholine (ACh), which is primarily mediated by IP_3_ [17,18,20,23,24].

There are conflicting hypotheses about the mechanism underlying NAADP-induced Ca^2+^ signalling [25]. Two-pore channels (TPC) [26,27] have been suggested as the main route for NAADP-induced Ca^2+^ release from the lysosomes. Calcium-induced calcium release (CICR) via IP_3_ or ryanodine receptors has been proposed as an explanation for the subsequent amplification of the response by Ca^2+^ release from the ER [26,27]. However, our group has emphasized the primary importance of ryanodine receptors for NAADP-induced Ca^2+^ release in both the secretory granule stores and the ER [22]. Any primary Ca^2+^ release from endosome/lysosome stores in pancreatic acinar cells would be extremely difficult to detect due to the small size of the endo/lysosomal Ca^2+^ store [15,28]. Currently, many aspects of NAADP-elicited Ca^2+^ signalling are very unclear [29,30] and it is therefore necessary to examine experimentally the different potential mechanisms [25,31]. Here we report a comprehensive comparison of the role of different types of RyRs and TPCs in NAADP-induced Ca^2+^ release in pancreatic acinar cells.

## 2 Materials and methods

### 2.1 Materials

Most of the reagents were purchased from Sigma (UK). Ned-19 was obtained from Tocris (UK). Thapsigargin and ATP were from Merck Millipore (UK). Rabbit anti-TPC1 and anti-TPC2 polyclonal antibodies were purchased from Lifespan BioSciences (UK). Rabbit anti-RyRs polyclonal antibodies were obtained from Merck Millipore (UK).

### 2.2 Pancreatic acinar cells isolation

Pancreatic acinar cells (single, or clusters of two or three cells) were isolated from the pancreas of adult C57BL/6 mice (control wt and mutant male or female mice with C57Bl/6JJmsSIc origin) as described previously [4]. Briefly, animals were killed according to UK Schedule 1 regulations and after dissection the pancreas was transferred into a collagenase-containing solution (200 U/ml, Worthington, UK), and incubated at 37 °C water bath for 14–15 min. After digestion, the tissue was mechanically disrupted in Na-Hepes based extracellular buffer, containing (mM): NaCl, 140; KCl, 4.7; Hepes KOH, 10; MgCl_2_, 1; glucose 10; CaCl_2_ 1; pH 7.2. Cells were then loaded with an appropriate fluorescent dye (Fluo-5N AM or Fluo-4 AM) following the manufacturer's instruction. All experiments were carried out at room temperature using freshly isolated cells, attached to the poly-L-lysine coverslips of the perfusion chamber.

### 2.3 Cell permeabilization protocol

Two-photon permeabilization was performed as described previously [22] using Leica multiphoton system SP5 with Mai-Tai two-photon laser tuned to 738 nm. Fluorescence intensity of Fluo-5N was recorded using excitation at 476 nm and emission >500 nm. During permeabilization and experimental protocols with permeabilized cells an intracellular solution was used based on K-Hepes, containing (mM): KCl, 127; NaCl, 20; Hepes KOH, 10; ATP, 2; MgCl_2_, 1; EGTA, 0.1; CaCl_2_ 0.05; pH 7.2.

### 2.4 Data analysis

To enable comparison, confocal images were recorded using identical laser power, photomultiplier sensitivity, and were processed using identical values for contrast and brightness. Statistical significance for the comparison between two groups of data Student's *t*-test was used (statistical significance was taken as *p* < 0.05).

## 3 Results

### 3.1 Inhibitory effect of NAADP antagonist Ned-19 on Ca^2+^ signal generation in pancreatic acinar cells

**Fig. 1:**
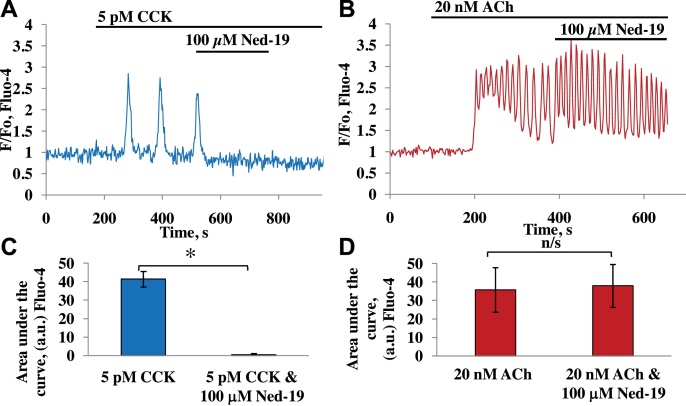
Ned-19 at high concentration (100 μM) considerably inhibited CCK responses in pancreatic acinar cells. A–B. Representative traces of responses to 5 pM CCK (A) or 20 nM ACh (B) followed by application of 100 μM Ned-19 in the continuous presence of CCK (A, *n* = 9) or ACh (B, *n* = 14). C–D. Quantification of the area under the curve (during 400 s from the addition point of 5 pM CCK or 100 μM Ned-19) in the experiments shown in A and B (41.3 ± 4.2 a.u. for 5 pM CCK and 0.5 ± 0.6 a.u. for 5 pM CCK and 100 μM Ned-19). Quantification of the area under the curve for ACh and Ned-19 is shown in D: 35.7 ± 12 a.u. for 20 nM ACh and 37.9 ± 11.6 a.u. for 20 nM ACh and 100 μM Ned-19 (D)). Error bars show ± SEM. *p < 4.9*10^−8^; n/s-non significant *p* > 0.78. Cells were loaded with Fluo-4 in AM form.

Previously it has been shown that Ca^2+^ responses elicited by physiological concentrations of CCK can be specifically blocked by inactivation of the NAADP receptor, while responses to ACh were unaffected [18,23]. The relationship between CCK action and NAADP signalling was shown by measurements of the intracellular level of NAADP that were increased in a dose-dependent manner upon stimulation with physiological concentrations of CCK. In contrast, ACh did not change the intracellular NAADP concentrations [17]. We employed the cell-permeable NAADP analogue and selective antagonist, Ned-19 [32] in order to explore differences between the mechanisms of ACh- and CCK-evoked Ca^2+^ signal generation. In a previous study, Ned-19, at a high concentration (100 μM), was shown to block NAADP-mediated Ca^2+^ release and binding to the NAADP receptor [33]. In our experiments on intact acinar cells, exposure of the cells to 100 μM Ned-19 was able to block completely cytosolic Ca^2+^ responses induced by 5 pM CCK (Fig. 1A and C), but was unable to inhibit Ca^2+^ oscillations produced by 20 nM ACh (Fig. 1B and D).

**Fig. 2:**
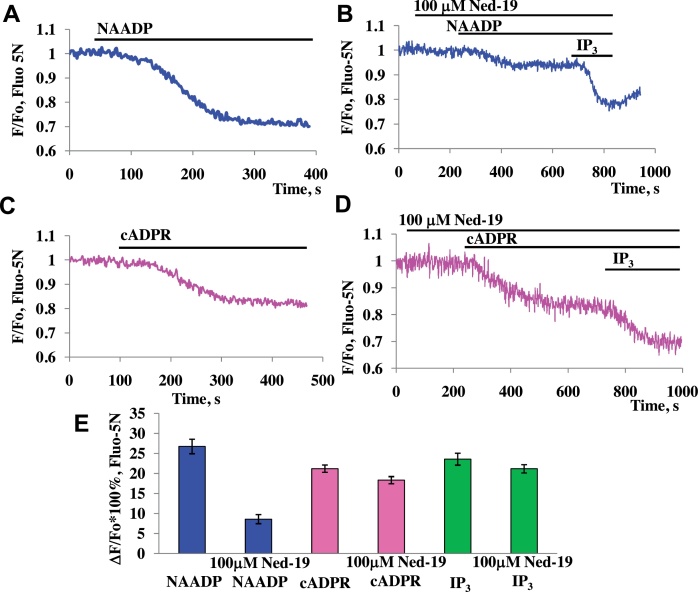
Inhibitory effect of Ned-19 (100 μM) on NAADP-induced signalling in permeabilized cells. (A) Representative trace of application of 100 nM NAADP to permeabilized pancreatic acinar cells. (B) Representative trace of application of 100 nM NAADP followed by 10 μM IP_3_ to permeabilized pancreatic acinar cells in the presence of 100 μM Ned-19. (C) Representative trace of application of 10 μM cADPR to permeabilized pancreatic acinar cells. (D) Representative trace of application of 10 μM cADPR followed by 10 μM IP_3_ to permeabilized pancreatic acinar cells in the presence of 100 μM Ned-19. (E) Summary of data comparison of applications of NAADP (control 26.7 ± 1.8%, *n* = 13; with Ned-19 8.6 ± 1.1, *n* = 5, *p* = 0.00002), cADPR (control 21.2 ± 0.9%, *n* = 10; with Ned-19 18.3 ± 0.9%, *n* = 4; *p* = 0.1) or IP_3_ (control 23.6 ± 1.5%, *n* = 11; with Ned-19 21.2 ± 1.1%, *n* = 6, *p* = 0.3) in the presence and absence of Ned-19. Error bars represent ± SEM. Cells were loaded with Fluo-5N in AM form.

In two-photon permeabilized pancreatic acinar cells, pre-incubation with a high concentration of Ned-19 (100 μM) inhibited NAADP-elicited Ca^2+^ responses by 67.8% (Fig. 2A, B, and E), whereas responses to cADPR (10 μM) (Fig. 2C–E) and IP_3_ (Fig. 2E) were not significantly affected, indicating that the action of Ned-19 is specific for NAADP-induced responses.

### 3.2 Both TPCs are involved in NAADP-elicited Ca^2+^ responses with predominant role of TPC2 in endosomes/lysosomes stores

**Fig. 3:**
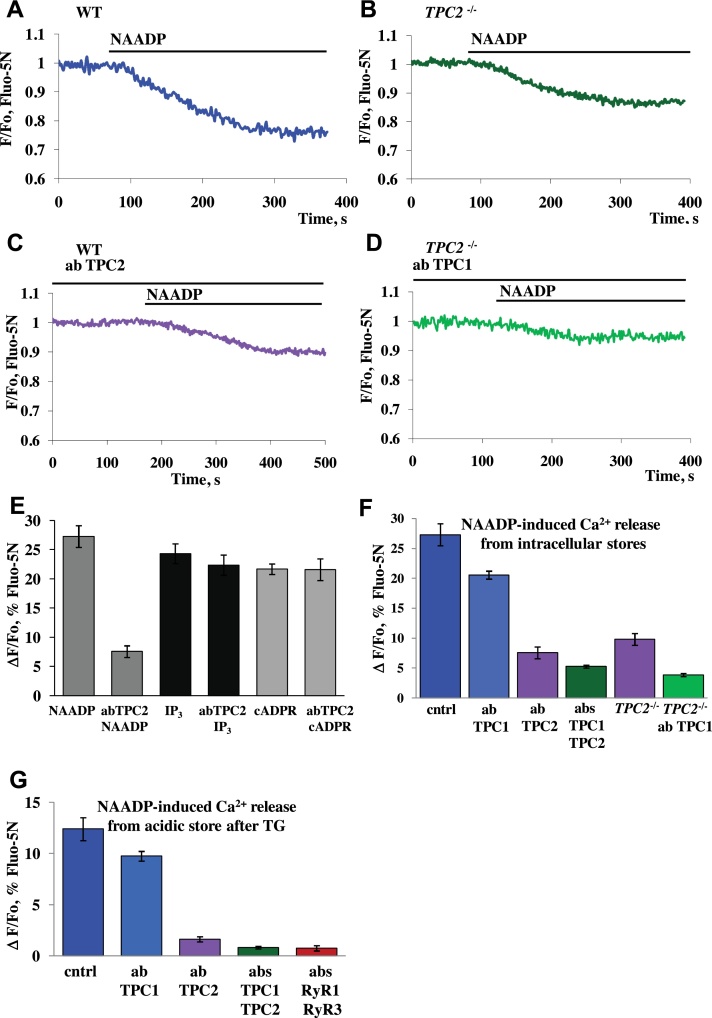
Involvement of TPC2 channels in NAADP-elicited Ca^2+^ release. (A) Trace represents an application of 100 nM NAADP to permeabilized cell isolated from wt mouse (*n* = 13). (B) Representative trace of application of 100 nM NAADP to permeabilized cell isolated from TPC2-KO mouse (*n* = 5). (C) Permeabilized cells from wt mice were pre-incubated with TPC2 antibody (20 min, 1:100) followed by addition of 100 nM NAADP (*n* = 8). (D) Representative trace shows typical response to 100 nM NAADP in permeabilized cells from TPC2-KO mice treated with TPC1 antibody (20 min, 1:100) (*n* = 11). (E) Comparison of the amplitudes of responses to 100 nM NAADP (*n* = 13 control; *n* = 8 with TPC2 antibody treatment), 10 μM IP_3_ (*n* = 9 control; *n* = 8 with TPC2 antibody treatment) or 10 μM cADPR (*n* = 9 control; *n* = 5 with TPC2 antibody treatment) in pre-treated or non-treated cells with antibody against TPC2. (F) Summary of NAADP-elicited Ca^2+^ release from the intracellular stores in permeabilized control cells or pre-treated with antibodies against TPC1 (20.6 ± 0.7%, *n* = 11), or TPC2 (7.6 ± 1%, SEM, *n* = 8), or mixture of both (5.3 ± 0.2%, *n* = 4), in wt mice compared to responses in permeabilized cells isolated from TPC2 KO mice and treated (3.9 ± 0.3%, *n* = 7) or non-treated with TPC1 antibody (9.8 ± 1.0%, *n* = 5). (G) Summary of results obtained from permeabilized cells treated with 10 μM thapsigargin to deplete ER followed by subsequent application of NAADP (100 nM) in the presence of antibodies against TPC1 (9.8 ± 0.5%, *n* = 5, *p* > 0.08), or TPC2 (1.63 ± 0.3%, *n* = 6), or a mixture of both (0.83 ± 0.1%, *n* = 6), or a mixture of antibodies against RyR1 and RyR3 (0.75 ± 0.1%, *n* = 5) as compared to control NAADP responses (12.4 ± 1.1%, *n* = 5). Error bars represent ± SEM. Cells were loaded with Fluo-5N in AM form.

Involvement of TPCs in NAADP-elicited Ca^2+^ signalling has been shown by many groups [26,34]. To further investigate this issue, we have performed experiments using pancreatic acinar cells isolated from TPC2 KO mice [26]. NAADP (100 nM) induced Ca^2+^ release from stores was reduced by 64% in cells from TPC2 KO as compared to the release in cells from wt mice (Fig. 3A, B, and F). A similar (72%) level of inhibition was achieved by treatment of permeabilized cells from wt mice with a TPC2 antibody (Fig. 3C and F). In contrast, an antibody against TPC1 was only able to reduce the response to a minor degree (by 24.5%) (Fig. 3F). Responses to cADPR and IP_3_ were not affected by the TPC2 antibody (Fig. 3E). Pre-incubation of permeabilized cells from TPC2 KO with the TPC1 specific antibody resulted in significant (*p* < 0.0002) inhibition of the NAADP-elicited Ca^2+^ release by 85.7% (Fig. 3D and F). Application of a mixture of both antibodies against TPC1 and TPC2 reduced NAADP-induced Ca^2+^ response by 80.6% (Fig. 3F).

These data indicate that both TPCs are involved in NAADP-induced Ca^2+^ signalling in pancreatic acinar cells, but also suggest that TPC2 is far more important than TPC1.

In endo-lysosomal stores TPC2 plays a particularly dominant role. After emptying specifically the ER stores (with 10 μM thapsigargin), the NAADP-elicited release of Ca^2+^ was inhibited by 86.9% by antibodies against TPC2, as compared to what was observed in wt cells (Fig. 3G). The NAADP-evoked Ca^2+^ release when an antibody against TPC1 was employed was not significantly different from the control (Fig. 3G). However, a combination of TPC1 and TPC2 antibodies resulted in a more substantial inhibition of the NAADP-induced Ca^2+^ signals, by 93%, as compared to the control level and this was similar to the effect induced by a mixture of antibodies against RyR1 and RyR3 (reduction by 94%) (Fig. 3G).

It has been reported that the P2 receptor antagonist pyridoxalphosphate-6-azophenyl-2′,4′-disulfonic acid (PPADS) can significantly inhibit NAADP-induced Ca^2+^-release in rat astrocytes and in the sea urchin egg [35,36]. It has been shown that it can compete with ATP, as a nucleotide mimetic, for a binding site on P2 receptors both in the rabbit vas deferens and the rabbit urinary bladder [37,38]. However, the precise mechanism and specificity of PPADS action on NAADP signalling is not fully understood. There are some indications in the literature demonstrating that PPADS may also affect other pathways including IP_3_ receptors [39]. We have pre-incubated permeabilized cells with 20 μM PPADS and applied 100 nM NAADP in the presence of this blocker that resulted in significant inhibition of NAADP-induced Ca^2+^ signals by 75.8% (6.6 ± 0.6%, *n* = 10). IP_3_ (10 μM)- or cADPR (10 μM)-induced Ca^2+^ responses were not reduced in the presence of 20 μM PPADS.

### 3.3 Involvement of RyRs in NAADP signalling

**Fig. 4:**
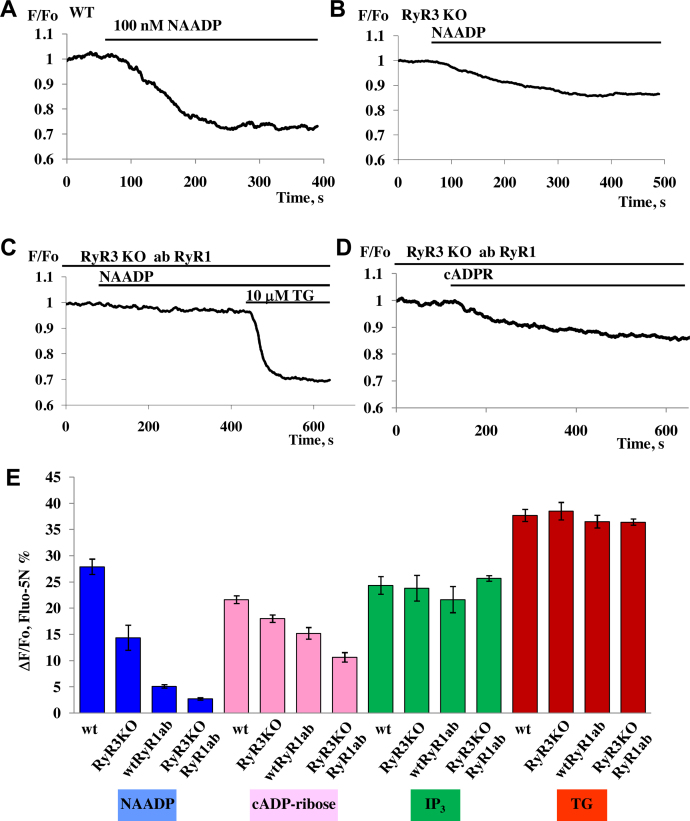
NAADP-elicited Ca^2+^ release from intracellular stores was reduced in pancreatic acinar cells isolated from RyR3 KO mice. (A) Control application of NAADP (100 nM) to permeabilized pancreatic acinar cells isolated from wt mice (*n* = 12). (B) Application of NAADP (100 nM) to permeabilized pancreatic acinar cells isolated from RyR3 KO mice (*n* = 8). (C) Permeabilized cells from RyR3 KO were incubated with RyR1 antibodies for 20 min (1:100) followed by subsequent applications of 100 nM NAADP and 10 μM thapsigargin (*n* = 10). (D) Permeabilized cells from RyR3 KO were incubated with RyR1 antibodies for 20 min (1:100) followed by subsequent applications of 10 μM cADPR and 10 μM thapsigargin (10.6 ± 0.9%, *n* = 9 as compared to control cADPR responses from wt 21.6 ± 0.7, *n* = 11). (E) Comparison of amplitudes of Ca^2+^ responses to NAADP (100 nM), IP_3_ (10 μM) and thapsigargin (10 μM) obtained using permeabilized cells from wt mice and RyR3 KO mice in the presence or absence of treatment with antibody against RyR1 (*n* > 4 for each group). Data represent mean values ± SEM. Cells were loaded with Fluo-5N AM.

To test for a role of RyRs in NAADP-induced Ca^2+^ signalling, we have employed permeabilized pancreatic acinar cells isolated from RyR3 KO mice. The NAADP (100 nM) elicited Ca^2+^ release from the internal stores of RyR3 KO cells was reduced to about 48% of the control value obtained from wt cells (Fig. 4A, B and E). These data strongly suggest that RyR3 is involved in NAADP-induced Ca^2+^ signalling. Pre-incubation of permeabilized cells isolated from RyR3^−/−^ cells with a RyR1 antibody almost abolished the NAADP responses (∼90% inhibition) (Fig. 4C and E). A similar protocol using RyR3 KO cells in the presence of RyR1 antibody was able to block cADPR-mediated Ca^2+^ release by only 49% (Fig. 4D and E). Responses to IP_3_ (10 μM) or thapsigargin (10 μM) in cells from RyR3 KO animals were not significantly different from responses in wt cells (Fig. 4E).

To test the involvement of different RyR sub-types in NAADP signalling, we used pre-incubation of permeabilized cells with antibodies against RyR1, 2 and 3. Supplemental Figures SP1A and SP1G show that pre-treatment of cells with antibodies against RyR1 dramatically decreased the responses to NAADP (100 nM) (5.1 ± 0.3%, *n* = 5) as compared to control (27.3 ± 1.9%, *n* = 12). Subsequent application of 10 μM IP_3_ resulted in a substantial Ca^2+^ release from the internal stores showing that the stores were duly loaded and capable of Ca^2+^ release (SP1A,G). The Ca^2+^ release elicited by cADPR (10 μM) in permeabilized cells pre-incubated with the RyR1 antibody was only modestly decreased (15.2 ± 1.1%, *n* = 5) as compared to control (21.7 ± 0.9%, *n* = 9) (SP1G).

Incubation of permeabilized cells with the RyR2 antibody did not significantly affect the magnitude of the NAADP-elicited Ca^2+^ release (SP1 C, SP1G), but the cADPR responses were substantially reduced (9.8 ± 0.7%, *n* = 5) (SP1D, SP1G). The RyR3 antibody partially reduced both cADPR and NAADP responses (SP1E, SP1F, SP1G).

Combination of antibodies against RyR1 and RyR2 markedly inhibited the NAADP and cADPR responses (4.75 ± 0.5%, *n* = 5 for NAADP; 3.6 ± 0.6%, *n* = 11 for cADPR) (SP1G). The largest inhibitory effect on NAADP-elicited Ca^2+^ release (nearly complete inhibition, 93%) was achieved when permeabilized cells were pre-treated with a combination of antibodies against RyR1 and RyR3 (1.9 ± 0.4%, *n* = 5) (SP1G). In the same set of experiments cADPR-induced responses were only partially inhibited (11 ± 0.6%, *n* = 5) (SP1G) whereas IP_3_-elicited Ca^2+^ release was unaffected by the RyRs antibodies (SP1G).

A mixture of antibodies against all three types of RyRs almost completely prevented both NAADP – and cADPR-induced Ca^2+^ release from intracellular stores of permeabilized cells (1.3 ± 0.2%, 93% inhibition, *n* = 5 for NAADP; 2.3 ± 0.9%, 89% inhibition, *n* = 5 for cADPR) (SP1B, SP1G). These data suggest that both NAADP- and cADPR-elicited Ca^2+^ signalling are dependent on functioning RyR channels in pancreatic acinar cells. As shown in Figure SP1G, the inhibition of RyR types 1 and/or 3 exhibits the most effective decrease of NAADP-induced signalling while inhibition of RyR2 has no effect. All three types of RyRs are involved in cADPR responses with RyR2s being the most important. IP_3_ (10 μM) in the presence of RyR and/or TPEN (200 μM) were used as a control of Ca^2+^ release from internal Ca^2+^ stores (SP1G).

**Fig. 5:**
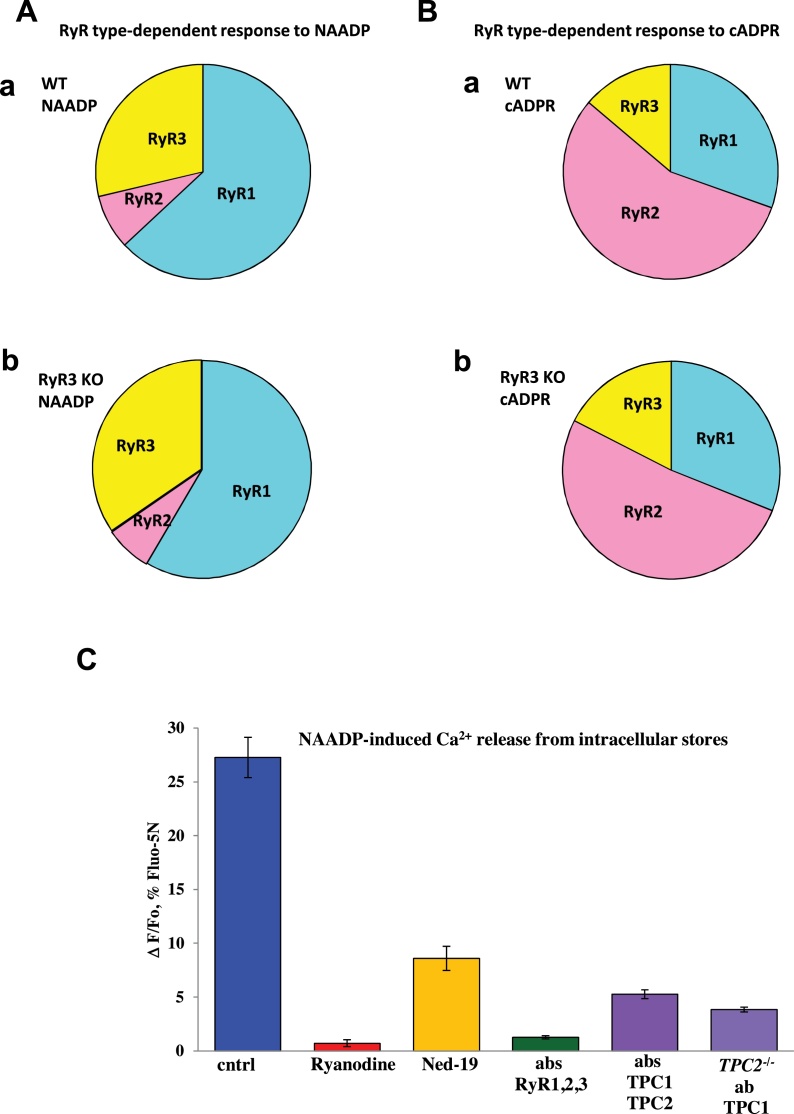
(A) Schematic distribution of RyRs type-dependent responses to 100 nM NAADP in pancreatic acinar cells from wt (a) or RyR3-KO (b) (based on knockouts and antibodies data shown in Fig. 4). (B) Schematic distribution of RyRs type-dependent responses to cADPR in pancreatic acinar cells from wt (a) or from RyR3-KO (b) (based on knockouts and antibodies data shown in Fig. 4). (C) Comparison of the relative importance of the RyRs and TPCs for the NAADP-induced Ca^2+^ responses in permeabilized pancreatic acinar cells. Ryanodine (100 μM) practically abolished responses to NAADP (0.71 ± 0.30%, *n* = 7) as compared to control (27.3 ± 1.8%, *n* = 12). Ruthenium Red (10 μM) also abolished responses to NAADP (0.33 ± 0.11, *n* = 6; not shown). Mixture of antibodies to all types of RyRs (RyR1 + RyR2 + RyR3) also dramatically reduced responses (1.27 ± 0.15%, *n* = 3). Specific inhibitor of NAADP-induced signalling Ned-19 has significantly reduced responses (8.6 ± 1.12%, *n* = 5). Mixture of antibodies to TPC1 and TPC2 reduced responses (5.3 ± 0.24%, *n* = 7). Antibodies to TPC1 applied to the cells isolated from TPC2 knockouts reduced responses (3.9 ± 0.27%, *n* = 7). Error bars show ± SEM. Cells were loaded with Fluo-5N in AM form before permeabilization.

Fig. 5 A and B presents schematically the relative importance of RyR sub-types for NAADP- and cADPR-induced Ca^2+^ release. By analysing the distribution of RyR type-dependent responses to 100 nM NAADP or cADPR in cells from wt and RyR3 KO mice in the presence or in the absence of RyR type specific antibodies (Fig. 4F and G) we have shown that RyR types 1 and 3 are most important for NAADP-elicited Ca^2+^ release, whereas RyR2 is mainly responsible for the cADPR-induced responses.

To compare the contribution of RyRs and TPCs, we used ryanodine or a mixture of antibodies to all three subtypes of RyRs (Fig. 5C) to inhibit RyRs and Ned19 or a mixture of antibodies to both TPCs (or antibodies to TPC1 in TPC2 knockouts) (Fig. 5C). Pre-treatment of permeabilized cells with 100 μM ryanodine resulted in almost complete inhibition of the NAADP responses, in agreement with our previous data [22], while the mixture of antibodies to all three types of RyRs inhibited responses by 95% (Fig. 5G). In contrast, Ned-19 inhibited the responses to NAADP to a lesser extent (68%) and a mixture of antibodies to TPC1 and TPC2 inhibited responses by 80%. When antibodies to TPC1 were applied to cells from TPC2 knockouts responses were reduced by 86% (Fig. 5C). Therefore, we conclude that inhibition of RyRs practically blocks the NAADP-induced Ca^2+^ release, whereas inhibition of TPCs results in a very significant reduction of the responses, but is unable to abolish the Ca^2+^ release. However, in acidic Ca^2+^ stores (Fig. 3G), antibodies to TPC1 and TPC2 essentially abolish the NAADP-induced Ca^2+^ responses, similarly to the mixture of antibodies to RyR1 and RyR3 (Fig. 3G).

## 4 Discussion

Our results provide fresh evidence for the importance of both TPCs and RyRs in NAADP-elicited Ca^2+^ signalling in pancreatic acinar cells. The results with the NAADP receptor antagonist Ned-19 (Fig. 1) confirm the conclusion from an earlier study [23], in which a high NAADP concentration was used to block NAADP receptors, indicating that operational NAADP receptors are required for cytosolic Ca^2+^ spiking evoked by physiological concentrations of CCK, whereas these receptors are not at all involved in Ca^2+^ spiking elicited by ACh. NAADP-mediated Ca^2+^ signalling is thus of importance for the normal function of pancreatic acinar cells. It is well established that repetitive Ca^2+^ spiking – irrespective of whether it is elicited by ACh or CCK or by intracellular application of IP_3_, cADPR or NAADP - is dependent on both operational IP_3_Rs and RyRs [18] and these findings, together with the direct demonstration that Ca^2+^ spiking can also be elicited simply by intracellular Ca^2+^ infusion [40], led to the conclusion that an initial small release of Ca^2+^ from intracellular stores mediated either by activation of IP_3_Rs or RyRs caused a much larger release from both IP_3_Rs and RyRs via the mechanism of Ca^2+^-induced Ca^2+^ release (CICR) [18]. Although it is impossible in intact cells to clearly separate components of Ca^2+^ release from IP_3_Rs and RyRs, this is possible in permeabilized cells [22] and we have exploited this feature in the present work. Our results show that operational TPCs are required for Ca^2+^ release elicited by NAADP, but not for Ca^2+^ liberation evoked by cADPR or IP_3_ (Figs. 2 and 3). This is in agreement with the accumulating evidence from several systems [34,41] that TPCs play a key role specifically in NAADP-mediated Ca^2+^ signalling. However, our results (Fig. 4 and SP1) also confirm our previous findings in permeabilized cells [22] that both NAADP- and cADPR-elicited Ca^2+^ release require functional RyRs, whereas this is not the case for IP_3_-evoked Ca^2+^ release.

Our new findings make it necessary to revise the current model [18] for CCK-evoked, NAADP-mediated Ca^2+^ signalling in pancreatic acinar cells. The simplest interpretation of our data suggests that the CCK-evoked rise in the intracellular NAADP concentration [17] primarily triggers opening of TPCs in an acid compartment, releasing a very small quantity of Ca^2+^, and that this Ca^2+^ release is sufficient to raise the local Ca^2+^ concentration in a micro-domain to activate RYRs via CICR. This could be envisaged as a very small primary Ca^2+^ release from an acid store (endosomes, lysosomes) being amplified very substantially by further Ca^2+^ release from both larger acid stores (zymogen granules) and the ER. This interpretation fits well with the previous finding that NAADP-elicited Ca^2+^ release from acid stores is blocked by clamping the cytosolic Ca^2+^ concentration at the normal resting level, whereas this is not the case for Ca^2+^ release evoked by cADPR and IP_3_ (Fig. 6 in [22]). It also fits well with our new finding that NAADP-elicited Ca^2+^ release from acid stores is completely dependent on functional TPCs (Fig. 3G). In this model, the RyRs are assigned a key role in CICR, as indeed they were in a much earlier model of ACh-evoked Ca^2+^ spiking [40]. Recent near-atomic resolution structures of RyR1 have finally given insights into the molecular mechanism of CICR [42,43].

Some of our new findings do, however, require some additions to the simple model outlined above. It is clear that whereas both NAADP- and cADPR-elicited Ca^2+^ release is completely dependent on RyRs (Fig. 5), the sub-type requirements are far from identical, as summarized in Fig. 5. The simplest way of reconciling these findings with our model would be to postulate different spatial distributions of NAADP and cADPR receptors, possibly even on different organelles. Since it would appear that cADPR can activate RyRs in a manner not requiring CICR [22], this could possibly indicate some more direct linkage to RyR2s. As the NAADP response from acid pools is particularly dependent on RyR1s (Fig. 5), and requires CICR [22], one could envisage that the NAADP receptors were particularly close to RyR1s, so as to allow CICR.

We have previously reached the conclusion that IP_3_Rs and RyRs are both present in acid pools and the ER and that all 3 intracellular Ca^2+^ releasing messengers can liberate Ca^2+^ from both pools [18,22]. Our new findings, showing that NAADP-elicited Ca^2+^ release from all the intracellular stores is not totally dependent on TPCs (Fig. 3F), as is the case for the acid stores (Fig. 3G), may indicate that NAADP in addition to activating TPCs, and in particular TPC2, can also interact more directly with RyRs in the ER as previously suggested for both pancreatic acinar [22] and T-cells [44,45]. This would also be in agreement with our previous finding that while NAADP-evoked Ca^2+^ release from acid stores can be blocked by clamping the cytosolic Ca^2+^ concentration at 100 nM, this is not the case for NAADP-elicited Ca^2+^ release from all the stores [22]. It seems, however, unlikely that there is a physiological role for the component of NAADP-elicited Ca^2+^ release that is independent of TPCs because, in the intact cells, Ned-19 completely blocked cytosolic Ca^2+^ spiking evoked by a physiological concentration of CCK (Fig. 1). In the permeabilized cells, the NAADP-elicited Ca^2+^ release from all the stores was markedly reduced but not abolished by Ned-19 (Fig. 2) in agreement with the incomplete block of Ca^2+^ release from all stores in the absence of functional TPCs (Fig. 3F) and in contrast to the complete dependency on TPCs for NAADP-elicited Ca^2+^ release from the acid store (Fig. 3G).

Overall, our data provide critical evidence for a central role of TPCs, and particularly TPC2, in NAADP-elicited Ca^2+^ signalling in pancreatic acinar cells, but also reveal a more complex pattern of potential interactions between different Ca^2+^ release channels in various compartments that require further investigation.

## Authors’ contribution

JVG, RMC and PEF did the experimental work and analyzed the data together with OVG. KM and MWS organized experimental work with RyR3-KO. JP provided TPC2–KO. OHP wrote the paper together with OVG and JVG.

## Conflict of interest

The authors declare that they have no conflict of interest.
