# Supplementary material for: Both RyRs and TPCs are required for NAADP-induced intracellular Ca2+ release
Source: Cell Calcium. 2015 Sep;58(3):237–45. doi: 10.1016/j.ceca.2015.05.005 (PMC4539342; doi:10.1016/j.ceca.2015.05.005)
Supplement: Supplementary file 1 [file mmc1.pptx]

## Slide 1
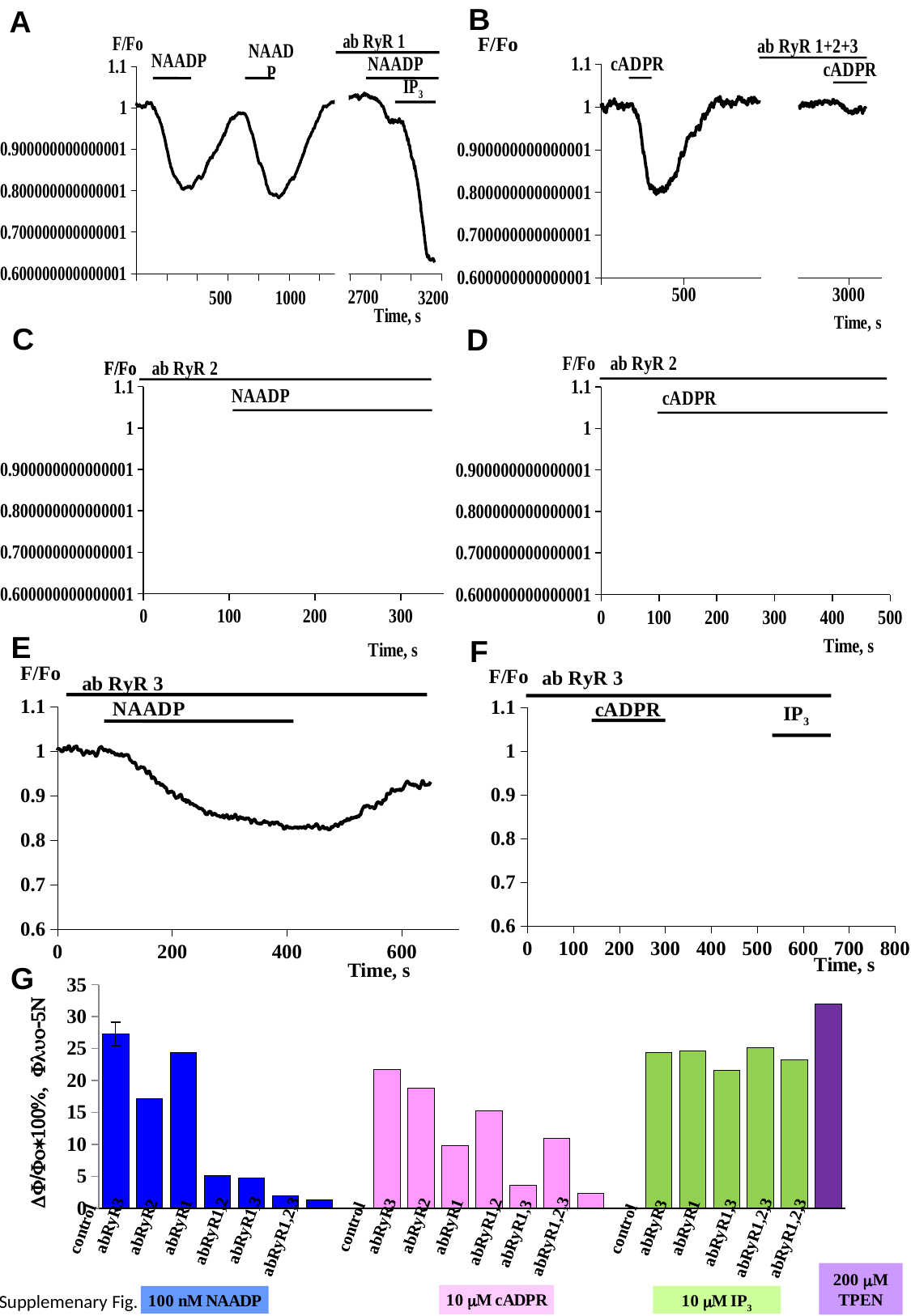

B
A
### Chart
| Category | | |
|---|---|---|F/Fo
### Chart
| Category | |
|---|---|C
D
### Chart
| Category | |
|---|---|
### Chart
| Category | |
|---|---|E
F
F/Fo
### Chart
| Category | |
|---|---|
### Chart
| Category | |
|---|---|Time, s
G
### Chart
| Category | | | | | | | | | | | | | | | | | | | | | |
|---|---|---|---|---|---|---|---|---|---|---|---|---|---|---|---|---|---|---|---|---|---|200 mM
TPEN
Supplemenary Fig.
